# Gut dysbiosis is associated with increased blood–brain barrier permeability and cognitive impairment in elderlies with coronary heart disease

**DOI:** 10.3389/fnagi.2025.1640761

**Published:** 2025-08-13

**Authors:** Lichao Di, Peiying Huang, Yeju He, Na Sun, Liwei Chi, Lining Huang

**Affiliations:** ^1^Department of Anesthesiology, The Second Hospital of Hebei Medical University, Shijiazhuang, China; ^2^Department of Medical Imaging, The Second Hospital of Hebei Medical University, Shijiazhuang, China; ^3^Hebei Key Laboratory of Neurodegenerative Disease Mechanism, Shijiazhuang, China; ^4^Key Laboratory of Clinical Neurology, Ministry of Education, Hebei Medical University, Shijiazhuang, China

**Keywords:** gut microbiota, gut-brain axis, blood–brain barrier, cognitive function, mild cognitive impairment, coronary heart disease, 16S rRNA sequencing, DCE-MRI

## Abstract

**Objective:**

The gut-brain axis is recognized as a critical pathway through which gut microbiota influences neurological health. However, the complex interplay between gut microbiota composition, blood–brain barrier (BBB) integrity, and cognitive function in elderly individuals with coronary heart disease (CHD) experiencing mild cognitive impairment (MCI) remains insufficiently elucidated. This study aimed to investigate these relationships in a cohort of 40 elderlies with CHD, comparing those with MCI to those with normal cognition (NC), focusing on microbial diversity, specific taxa alterations, BBB permeability, and their correlations with cognitive performance.

**Methods:**

This preplanned secondary analysis utilized data from two prospective cohort studies, enrolling elderlies with CHD (≥60 years). Participants were categorized into NC (*n* = 20) and MCI (*n* = 20) groups based on standardized neuropsychological assessments. Fecal samples underwent 16S rRNA gene sequencing (V3-V4 region) to evaluate gut microbiota diversity and composition. BBB permeability was quantified using dynamic contrast-enhanced magnetic resonance imaging (DCE-MRI), specifically measuring the volume transfer constant (Ktrans) in the hippocampus.

**Results:**

Compared to the NC group, MCI patients exhibited significantly reduced gut microbial *α*-diversity (Chao1 index: *p* = 0.002; Shannon index: *p* = 0.009) and distinct *β*-diversity profiles (Bray–Curtis dissimilarity, PERMANOVA, *p* = 0.003). LEfSe analysis identified depletion of key short-chain fatty acid (SCFA)-producing taxa in the MCI group, including at the family level (Ruminococcaceae, *p* = 0.016; Rikenellaceae, *p* = 0.042; and Barnesiellaceae, *p* = 0.038) and genus level (Faecalibacterium, *p* = 0.003 and Oscillospira, *p* = 0.002). Hippocampal BBB permeability (Ktrans) was significantly elevated in MCI patients (6.04 ± 3.02 vs. 3.90 ± 1.03 × 10^−3^ min^−1^, *p* = 0.006) and inversely correlated with the relative abundance of Faecalibacterium (Spearman’s r = −0.466, *p* = 0.002) and Oscillospira (Spearman’s r = −0.322, *p* = 0.043). Conversely, these genera showed positive correlations with Montreal Cognitive Assessment-Basic (MoCA-B) scores (Faecalibacterium: r = 0.596, *p* < 0.001; Oscillospira: r = 0.369, *p* = 0.019).

**Conclusion:**

Elderlies with CHD and MCI demonstrate significant gut dysbiosis, characterized by reduced microbial diversity and depletion of SCFA-producing taxa, notably butyrate producers. These microbial alterations are correlated with increased BBB permeability in the hippocampus and diminished cognitive function. These findings highlight the potential role of the gut-brain axis in the pathogenesis of cognitive decline in this vulnerable population and suggest that targeting gut microbiota could be a therapeutic avenue.

## Introduction

Alzheimer’s disease (AD) typically progresses through distinct stages, including a preclinical phase, mild cognitive impairment (MCI), and ultimately, dementia (Sperling et al., 2011). MCI represents a transitional state characterized by a noticeable decline in memory or other cognitive domains that does not substantially interfere with daily activities or meet the criteria for dementia (Petersen et al., 2018). As a critical prodromal stage of dementia, particularly AD, MCI offers a crucial window for early detection, diagnosis, and potential preventive interventions (Jia et al., 2020).

Emerging evidence implicates blood–brain barrier (BBB) breakdown as an early biomarker in the trajectory of cognitive dysfunction, including MCI and AD (Nation et al., 2019). The BBB, a highly selective semipermeable endothelial barrier, is crucial for maintaining brain homeostasis. Its disruption can lead to the influx of neurotoxic substances and peripheral immune cells, exacerbating neuroinflammation and neuronal damage (Kaddoumi et al., 2022). Consequently, strategies aimed at preserving or restoring BBB integrity are being explored as potential therapeutic approaches for MCI and AD (Kaddoumi et al., 2022; Wang et al., 2022).

The gut microbiome, the vast community of microorganisms residing in the gastrointestinal tract, modulates brain function and health via the gut-brain axis—a complex, bidirectional communication network (Borrego-Ruiz and Borrego, 2024). A diverse and stable gut microbiota is essential for overall host health, influencing metabolism, immunity, and neurodevelopment (Spielman et al., 2018). Recent research suggests that targeting the BBB through gut microbiota modulation could be a novel strategy to delay age-associated neurological diseases (Prajapati et al., 2025). In neurodegenerative conditions, alterations in the gut microbiota, termed dysbiosis, may contribute to neuroinflammation and compromise BBB integrity (Cryan et al., 2019; Kulkarni et al., 2025). One key mechanism involves microbial metabolites, particularly short-chain fatty acids (SCFAs) like butyrate, propionate, and acetate, which are produced from dietary fiber fermentation. These SCFAs are known to regulate immune responses, reduce inflammation, and enhance BBB integrity by upregulating tight junction protein expression (Silva et al., 2020; Martin-Gallausiaux et al., 2018). However, much of the evidence linking gut microbiota, SCFAs, and BBB integrity stems from preclinical animal models. Coronary heart disease (CHD) is a major risk factor for cognitive decline and dementia, likely through mechanisms including chronic cerebral hypoperfusion, systemic inflammation, and shared vascular risk factors (Deckers et al., 2017). The gut microbiome, in turn, is deeply implicated in cardiometabolic health. Dysbiosis can promote the development of CHD through pathways involving pro-inflammatory metabolites like trimethylamine N-oxide (TMAO) and reduced production of beneficial short-chain fatty acids (SCFAs), which have systemic anti-inflammatory effects (Chen X. et al., 2023). Studies in human populations, particularly those with comorbidities like CHD, which itself is linked to both cognitive decline and gut dysbiosis (Tang et al., 2017), are limited.

Elderly individuals with CHD represent a population at increased risk for cognitive decline. Understanding the interplay between gut microbiota, BBB permeability, and cognitive status in this specific group is crucial. Therefore, this study aimed to investigate the associations between gut microbiota composition, BBB permeability (quantified by dynamic contrast-enhanced MRI in the hippocampus), and cognitive function in elderlies with CHD with and without MCI. We hypothesized that elderlies with CHD and MCI would exhibit distinct gut microbial profiles, increased BBB permeability, and that these alterations would correlate with the severity of cognitive impairment.

## Materials and methods

### Study design and ethical approval

This study was a preplanned secondary analysis of data derived from two prospective cohort studies conducted at The Second Hospital of Hebei Medical University. The original studies focused on perioperative neurocognitive disorders and related biomarkers. The comprehensive data collection, including neuropsychological assessments, DCE-MRI for BBB evaluation, and fecal sample collection for microbiota analysis, was an integral part of the parent study protocols, designed prospectively to investigate the multifactorial nature of cognitive decline in this patient population. The rationale for utilizing these cohorts for the current analysis was the comprehensive collection of cognitive, imaging, and biospecimen data relevant to the gut-brain axis. Ethical approval for this secondary analysis and the parent studies was obtained from the Research Ethics Committee of The Second Hospital of Hebei Medical University (approval no. 2023-R077). Both parent studies were registered on the Chinese Clinical Trial Registry (ChiCTR2200063774 and ChiCTR2300070477). All participants provided written informed consent prior to enrollment.

### Participant enrollment

Participants were recruited between April 2023 and August 2023. Inclusion criteria were: (1) age between 60 and 80 years; (2) a minimum of 6 years of education; (3) American Society of Anesthesiologists (ASA) physical status grade II–III; and (4) a diagnosis of coronary heart disease (CHD), confirmed by coronary angiography. Exclusion criteria included: (1) history of stroke, brain tumor, brain surgery, Parkinson’s disease, or epilepsy; (2) severe auditory or visual impairments, or communication difficulties hindering neuropsychological testing; (3) pre-existing major psychiatric disorders (e.g., schizophrenia, major depressive disorder requiring hospitalization); (4) contraindications for magnetic resonance imaging (MRI) scanning (e.g., metallic implants, claustrophobia); (5) active lower gastrointestinal tract diseases (e.g., inflammatory bowel disease), or acute/chronic infectious diseases; (6) use of antibiotics for more than 3 consecutive days within one month prior to enrollment, or regular use of probiotics/prebiotics. Each participant underwent a comprehensive medical history evaluation, detailed neuropsychological assessment, fecal sample collection, and brain MRI.

### Clinical and neuropsychological assessment

Demographic characteristics (age, sex, body mass index [BMI], education level), lifestyle factors (smoking, alcohol consumption), history of COVID-19 infection, and comorbidities (hypertension, diabetes, hypercholesterolemia, constipation, anxiety, New York Heart Association [NYHA] class, Pittsburgh Sleep Quality Index [PSQI]) were collected by trained researchers using standardized questionnaires.

A comprehensive neuropsychological test battery was administered by trained psychometrists in a quiet, controlled environment. Global cognitive function was assessed using the Montreal Cognitive Assessment-Basic (MoCA-B). Specific cognitive domains evaluated included: memory (Auditory Verbal Learning Test-Hua Shan version [AVLT-H], long-delayed free recall); executive function (Trail Making Test A [TMT-A]); language (Verbal Fluency Test [VFT], animal and fruit categories); and visuoconstructive skills (Clock Drawing Test-30 [CDT-30]). Activities of Daily Living (ADL) were assessed to evaluate functional independence, and the Clinical Dementia Rating Scale (CDR) was used to stage dementia severity (though primarily to exclude dementia). All scales were validated Chinese versions.

Participants were classified into two groups: normal cognition (NC) and mild cognitive impairment (MCI). The diagnosis of MCI was based on the criteria proposed by Albert et al. (2011), (1) subjective cognitive complaint by the patient or an informant; (2) objective evidence of impairment in one or more cognitive domains, defined as a score ≥1.5 SD below age-and education-adjusted norms on any of the domain-specific tests (AVLT-H, TMT-A, VFT, CDT-30), with norms derived from published Chinese population data; (3) largely preserved independence in daily living activities; and (4) absence of dementia. While the MoCA-B was used to assess global function, a specific cut-off score was not used in isolation for diagnosis due to its use as a screening tool.

### Blood–brain barrier permeability assessment

Brain imaging was conducted using a 3.0 Tesla GE SIGNA Architect MRI system (GE Healthcare). For anatomical localization, 3D T1-weighted BRAVO and 3D T2-weighted FLAIR CUBE sequences were acquired. The integrity of the BBB was evaluated through dynamic contrast-enhanced magnetic resonance imaging (DCE-MRI). The DCE-MRI acquisition parameters were as follows: axial plane, repetition time (TR) of 4.4 ms, echo time (TE) of 1.6 ms, slice thickness of 2.0 mm with a 0.6 mm interslice gap, field of view (FOV) measuring 26.9 cm × 24 cm, and a flip angle of 12°. A gadolinium-based contrast agent (Gadobutrol, 0.1 mmol/kg of body weight) was administered intravenously using a power injector at a flow rate of 4.5 mL/s, succeeded by a 20 mL saline flush. This injection commenced at the start of the third dynamic acquisition phase. In total, 40 dynamic scans were obtained over a period of approximately 7 min.

The acquired DCE-MRI datasets were subsequently processed on a GE Advantage Workstation (AW4.7) utilizing the GEN IQ advanced analysis module, which includes functionality for 3D motion correction. To quantify BBB permeability, the extended Tofts two-compartment pharmacokinetic model was employed, generating the volume transfer constant (Ktrans, units: min^−1^). An individual arterial input function (AIF) was determined for each participant from the M2 segment of the middle cerebral artery, initially identified automatically by the software and then meticulously reviewed and confirmed by an experienced neuroradiologist for accurate placement and characteristic curve shape. T1 mapping was carried out to correct for T1 relaxation times. For this study’s focus, regions of interest (ROIs) were specifically delineated in the bilateral hippocampi. This manual delineation was performed on co-registered T2-FLAIR images by two skilled neuroradiologists, who were kept unaware of the participants’ cognitive diagnoses and group assignments; any discrepancies in delineation were resolved through mutual agreement. The mean Ktrans values derived from these bilateral hippocampal ROIs served as the primary measure for subsequent statistical analysis.

### Fecal sample collection and 16S rRNA gene sequencing

Fecal specimen collection was conducted within the hospital setting. Participants were provided with standardized collection kits and clear instructions. Samples were typically obtained in the morning prior to major study-related procedures or assessments, and subsequently as defined by the study protocol. Upon collection, specimens were immediately refrigerated at 4°C. They were then transported to the laboratory facilities on dry ice, generally within a 2 h timeframe, and subsequently archived at −80°C pending DNA extraction. Efforts were made to coordinate fecal sample collection to occur within 48 h of the cognitive and imaging assessments.

Total microbial genomic DNA was extracted from approximately 200 mg of fecal material using an Omega Soil DNA Kit according to the manufacturer’s instructions. DNA concentration and purity were assessed using a NanoDrop spectrophotometer (Thermo Fisher Scientific, Waltham, MA, United States). The V3-V4 hypervariable regions of the 16S rRNA gene were amplified by PCR using specific primers (F: ACTCCTACGGGAGGCAGCA, R: GGACTACHVGGGTWTCTAAT) via PCR. PCR products were purified and pooled. Sequencing was performed on an Illumina NovaSeq 6,000 platform (Illumina, San Diego, CA, USA) by Personalbio Technology Co., Ltd. (Shanghai, China), generating 2×250 bp paired-end reads.

Raw sequencing reads were processed using QIIME2 (version 2022.2) (Bolyen et al., 2019). Briefly, reads were demultiplexed, followed by quality filtering, denoising, merging of paired-end reads, and chimera removal using the DADA2 plugin to generate amplicon sequence variants (ASVs). To ascertain the taxonomic composition, the representative sequences derived from the ASV generation step were subjected to a classification process within the QIIME 2 (version 2022.2) environment. This procedure involved querying these sequences against the Greengenes 16S rRNA gene reference database (release 13.8), enabling the assignment of taxonomic labels from phylum to genus levels based on sequence similarity.

### Statistical analysis

Statistical analyses were performed using SPSS (version 27.0; IBM Corp, Armonk, NY, United States) and R software (version 4.3.1; R Core Team, Vienna, Austria). Data distribution was assessed using Shapiro–Wilk tests. Continuous variables were expressed as mean ± standard deviation (SD) for normally distributed data or median [interquartile range (IQR)] for non-normally distributed data. Categorical data were presented as numbers (proportions, %). Group comparisons for continuous variables were made using independent samples t-tests or Mann–Whitney U tests, as appropriate. Categorical data were compared using Chi-squared (χ^2^) tests or Fisher’s exact test. A *p*-value < 0.05 (two-sided) was considered statistically significant.

For gut microbiota analysis: Alpha diversity indices (Chao1 for richness, Shannon for richness and evenness, Pielou’s e for evenness) were calculated based on ASV counts and compared between groups using Mann–Whitney U tests. Beta diversity was assessed using Bray–Curtis dissimilarity and visualized with Principal Coordinates Analysis (PCoA). Permutational multivariate analysis of variance (PERMANOVA) with 999 permutations was used to test for significant differences in overall microbiota composition between groups. Linear discriminant analysis Effect Size (LEfSe) was employed to identify differentially abundant taxa (from phylum to genus level) between the NC and MCI groups, using a logarithmic LDA score threshold of >2.0 and a Kruskal-Wallis test *p*-value < 0.05. Correlations between microbial taxa (relative abundances of genera identified as significant by LEfSe), hippocampal Ktrans values, and MoCA-B scores were assessed using Spearman’s rank correlation coefficients.

Sample size was initially estimated based on expected differences in hippocampal Ktrans values between NC and MCI patients from pilot data or literature review. The pilot trial indicated a mean Ktrans of 6.0 × 10^−3^ min^−1^ in MCI patients and 3.5 × 10^−3^ min^−1^ in NC patients. With a significance level (*α*) of 0.05 and a power (1-*β*) of 0.80, the required sample size was calculated to be 20 participants per group.

## Results

### Participant demographics and clinical characteristics

The final analysis included 40 participants: 20 in the NC group and 20 in the MCI group. Demographic and clinical characteristics are presented in Table 1. There were no significant differences between the NC and MCI groups in terms of age, sex distribution, BMI, smoking or drinking habits, education level, history of COVID-19 infection, or prevalence of hypertension, diabetes, hypercholesterolemia, NYHA class, constipation, or anxiety symptoms (PSQI scores). Additionally, key cardiometabolic markers and medication use were well-balanced between the groups (Table 1). This similarity in baseline characteristics helps to minimize their confounding effects on the primary outcomes.

**Table 1:** Patient characteristics and perioperative data.

Demographics	NC (*n* = 20)	MCI (*n* = 20)	*p*-value
Age, y, mean (SD)	69.00 (3.45)	68.40 (3.19)	0.571
Female, *n* (%)	6 (30)	5 (25)	0.723
BMI, kg/m^2^, mean (SD)	24.79 (2.42)	24.75 (3.16)	0.968
Smoking, *n* (%)	4 (20)	6 (30)	0.716
Drinking, *n* (%)	7 (35)	6 (30)	0.736
College education, *n* (%)	5 (25)	3 (15)	0.695
COVID-19 history, *n* (%)	8 (40)	10 (50)	0.525
Hypertension, *n* (%)	13 (65)	17 (85)	0.273
Diabetes, *n* (%)	6 (30)	8 (40)	0.507
Hypercholesterolemia, *n* (%)	7 (35)	5 (25)	0.731
NYHA (II/III), *n* (%)	8/12 (40/60)	7/13 (35/65)	0.744
Constipation, *n* (%)	4 (20)	7 (35)	0.298
Anxiety, *n* (%)	6 (30)	4 (20)	0.716
PSQI, mean (SD)	5.10 (3.73)	6.65 (4.42)	0.238
SBP, mmHg, mean (SD)	134.6 (10.5)	135.5 (9.3)	0.788
DBP, mmHg, mean (SD)	84.2 (6.4)	84.7 (6.8)	0.813
Fasting Glucose, mmol/L, mean (SD)	5.9 (0.6)	5.7 (0.6)	0.390
LDL-C, mmol/L, mean (SD)	2.9 (0.5)	3.0 (0.6)	0.590
Statins, *n* (%)	16 (80)	18 (90)	0.661
Antiplatelets, *n* (%)	18 (90)	19 (95)	1.000
PPIs, *n* (%)	1 (5)	2 (10)	1.000
Metformin, *n* (%)	1 (5)	3 (15)	0.605

Data are presented as mean (SD) or *n* (%). BMI, body mass index; COVID-19, Corona Virus Disease 2019; PSQI, Pittsburgh Sleep Quality Index; NYHA, New York Heart Association; SBP, Systolic Blood Pressure; DBP, Diastolic Blood Pressure; LDL-C, Low-Density Lipoprotein Cholesterol; PPIs, Proton-Pump Inhibitors.

### Neuropsychological assessment and BBB permeability

As expected, patients in the MCI group exhibited significantly lower scores on the MoCA-B compared to the NC group (19.05 ± 1.19 vs. 25.25 ± 1.65, *p* < 0.001). Significant differences were also observed in AVLT-H long-delayed recall, TMT-A completion time, and CDT-30 scores, indicating poorer performance in memory, executive function, and visuoconstructive skills in the MCI group (Table 2). There was a trend towards lower VFT scores in the MCI group, though it did not reach statistical significance (*p* = 0.059).

**Table 2:** Differences in cognitive scale scores and hippocampal Ktrans between groups.

Variable	NC (*n* = 20)	MCI (*n* = 20)	*p*-value
MoCA-B, mean (SD)	25.25 (1.65)	19.05 (1.19)	<0.001
AVLT-H (long-delayed recall), mean (SD)	6.65 (0.99)	5.85 (1.31)	0.035
TMT-A (seconds), mean (SD)	81.10 (8.99)	87.45 (6.15)	0.013
VFT (total score), mean (SD)	9.30 (1.75)	8.21 (1.82)	0.059
CDT-30, mean (SD)	21.60 (2.30)	19.90 (2.65)	0.037
Hippocampal Ktrans (×10^−3^ min^−1^), mean (SD)	3.90 (1.03)	6.04 (3.02)	0.006

Data are presented as mean (SD). MoCA-B, Montreal Cognitive Assessment-Basic; AVLT-H, Auditory Verbal Learning Test-Hua Shan version; TMT-A, Trail Making Test Part A; VFT, Verbal Fluency Test; CDT-30, Clock Drawing Test (30-point scoring); Ktrans, volume transfer constant.

Hippocampal BBB permeability, assessed by Ktrans values, was significantly higher in the MCI group (median 6.04 ± 3.02 × 10^−3^ min^−1^) compared to the NC group (median 3.90 ± 1.03 × 10^−3^ min^−1^; *p* = 0.006).

### Alterations in gut microbiota composition and diversity

**Figure 1:**
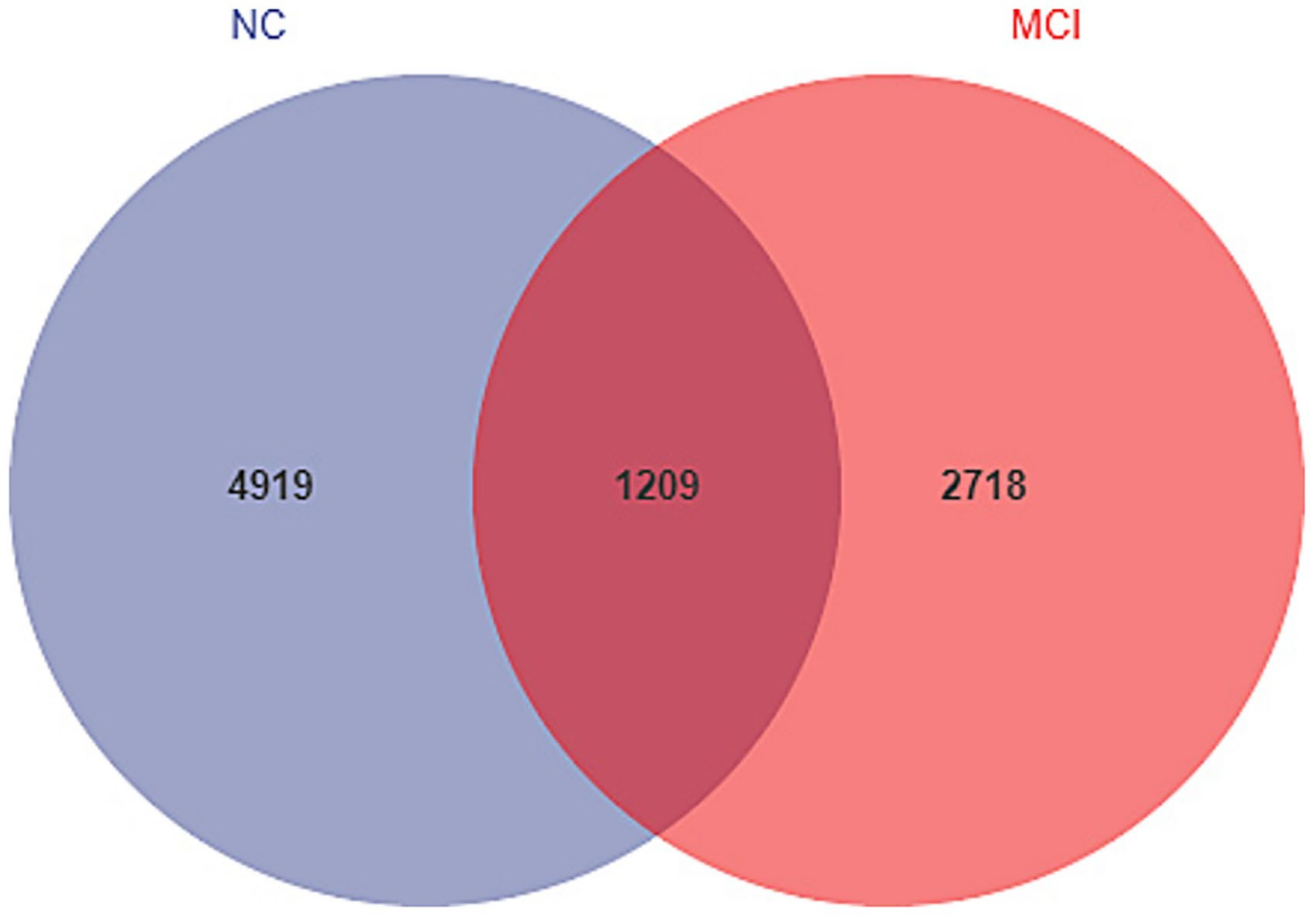
Venn diagram illustrating unique and shared amplicon sequence variants (ASVs) between the normal cognition (NC) and mild cognitive impairment (MCI) groups. Number of ASVs are indicated.

Rarefaction curve analysis indicated sufficient sequencing depth to capture the majority of bacterial diversity in all samples (Supplementary Figure 1). The Venn diagram of shared and unique ASVs revealed a lower number of observed ASVs in the MCI group compared to the NC group (Figure 1: 3927 unique/shared ASVs in MCI vs. 6,128 in NC).

**Figure 2:**
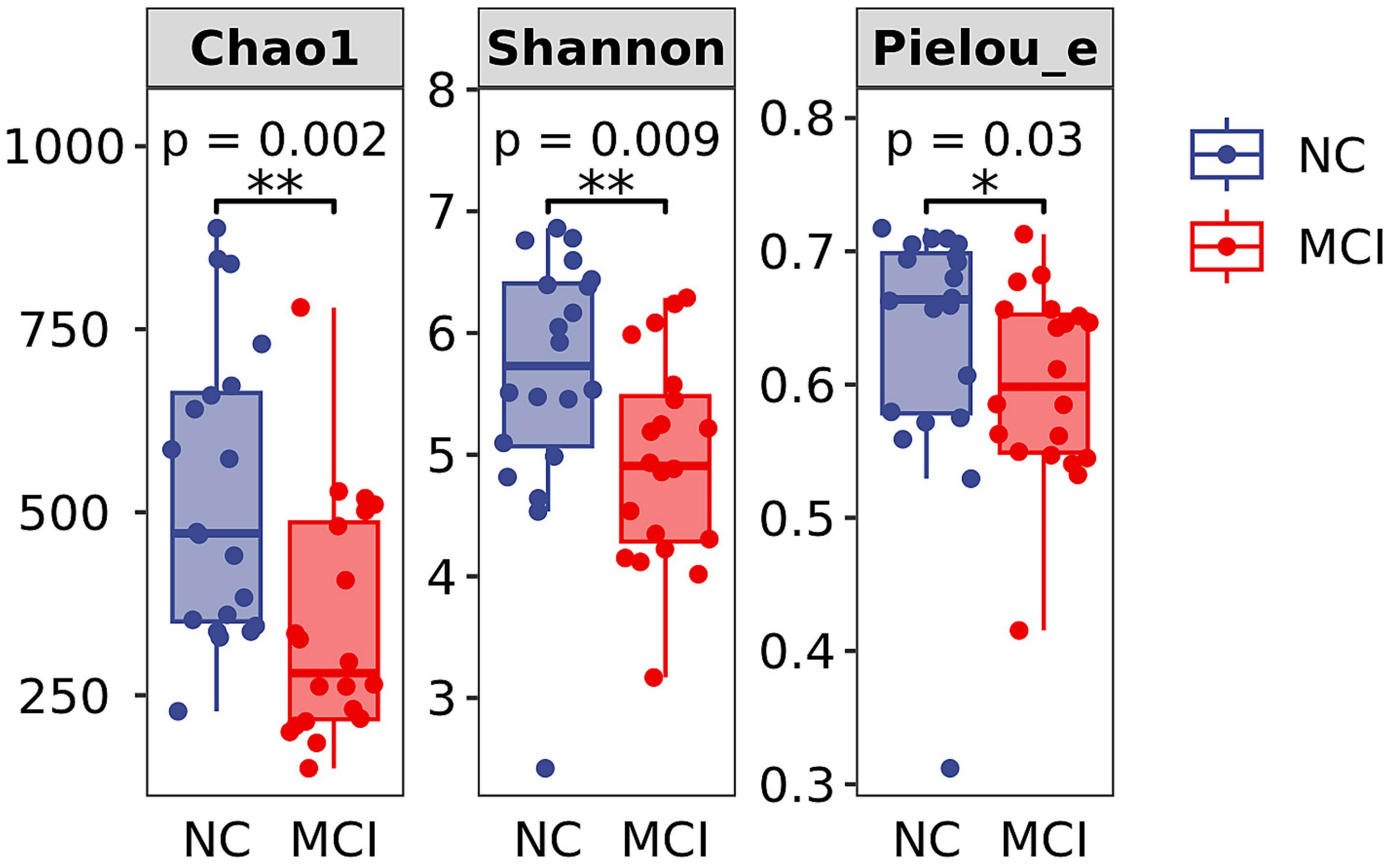
Alpha diversity indices of gut microbiota. Box plots showing Chao1 index, Shannon index, and Pielou’s evenness index in the Normal Cognition (NC) and Mild Cognitive Impairment (MCI) groups. The central line indicates the median, the box represents the interquartile range (IQR), and the whiskers extend to 1.5 times the IQR. Dots represent individual data points. Significant differences are indicated by *p < 0.05, **p < 0.01.

Alpha diversity analysis showed significantly lower microbial richness and diversity in the MCI group compared to the NC group. Specifically, the Chao1 index (343.9 ± 162.2 vs. 524.5 ± 199.3; *p* = 0.002), Shannon index (4.94 ± 0.84 vs. 5.64 ± 1.06; *p* = 0.009), and Pielou’s evenness index (0.60 ± 0.07 vs. 0.63 ± 0.10; *p* = 0.030) were all significantly reduced in MCI patients (Figure 2).

**Figure 3:**
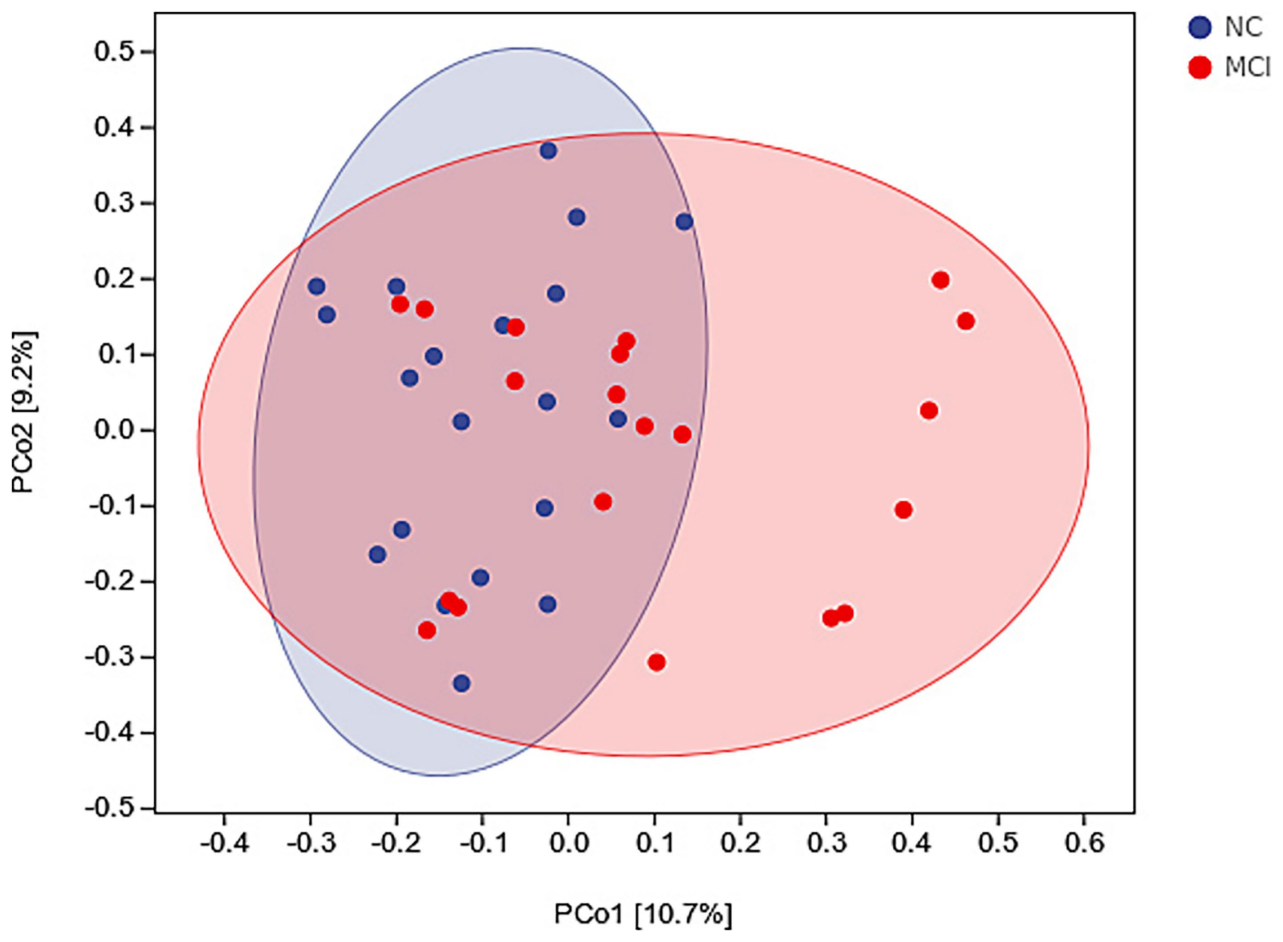
Beta diversity of gut microbiota. Principal Coordinates Analysis (PCoA) plot based on Bray–Curtis dissimilarity, illustrating differences in overall microbial composition between normal cognition (NC; blue dots) and mild cognitive impairment (MCI; red dots) groups. PERMANOVA: *p* = 0.003.

Beta diversity analysis using PCoA based on Bray–Curtis dissimilarity revealed a distinct clustering of gut microbiota composition between the NC and MCI groups (Figure 3). PERMANOVA confirmed this significant difference in overall microbial structure (*p* = 0.003).

### Differential abundance of gut microbial taxa

At the phylum level, Firmicutes and Bacteroidetes were the most abundant phyla across all samples, accounting for over 65% of the total sequences. We compared the Firmicutes-to-Bacteroidetes (F/B) ratio between the groups but found no statistically significant difference (*p* = 0.609), suggesting that broad phylum-level shifts were not a primary feature of dysbiosis in this cohort. The LEfSe analysis also did not identify any phyla as being differentially abundant between the groups.

**Figure 4:**
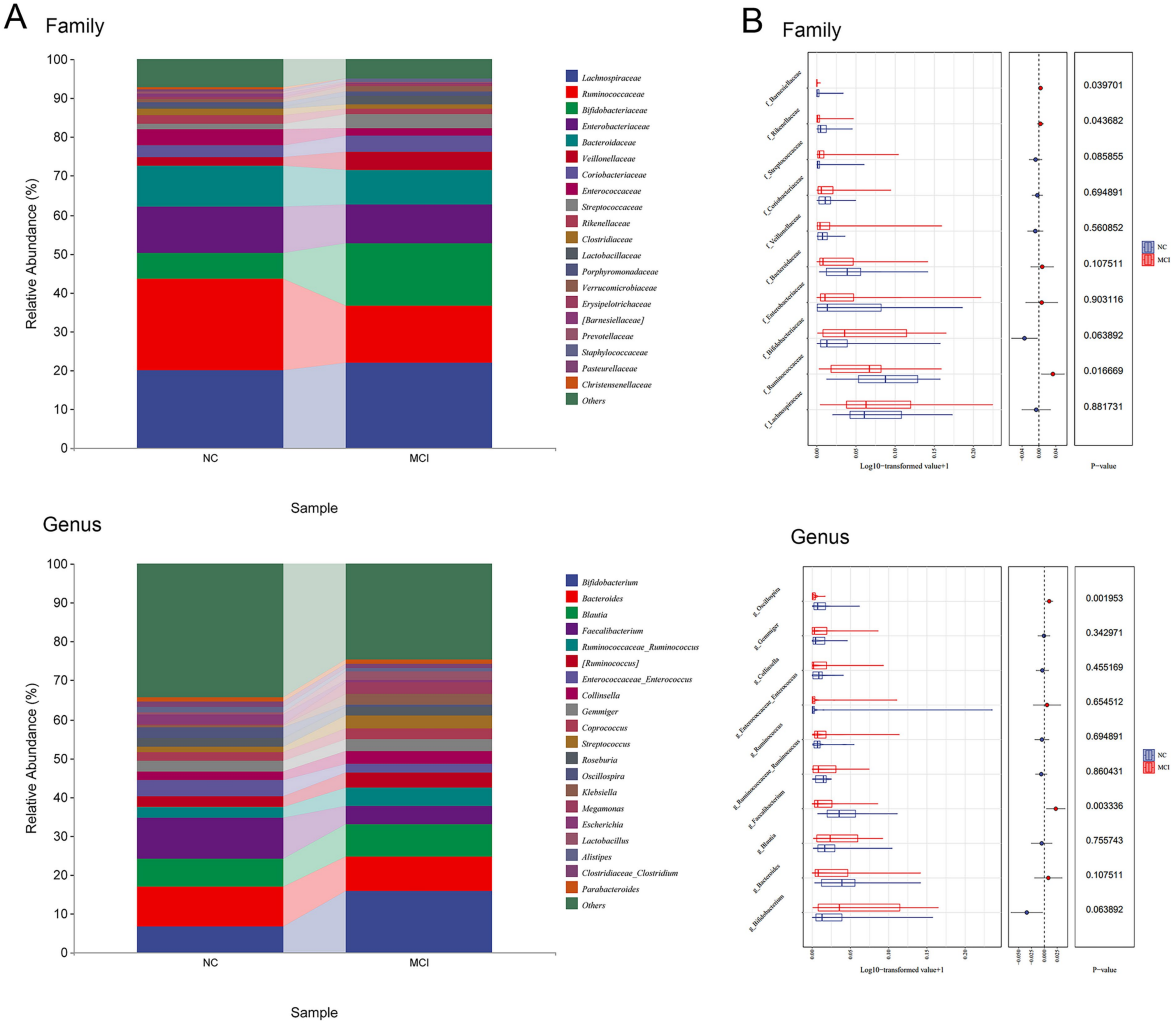
Differentially abundant gut microbial taxa between normal cognition (NC) and Mild cognitive impairment (MCI) groups identified by LEfSe analysis. **(A)** Microbial composition at family and genus levels. **(B)** Lefse analyses of microbial relative abundance.

LEfSe analysis identified several taxa that were differentially abundant between the NC and MCI groups (Figure 4). At the family level, the relative abundances of Ruminococcaceae (LDA score = 4.661, *p* = 0.016), Rikenellaceae (LDA score = 3.752, *p* = 0.042), and Barnesiellaceae (LDA score = 3.698, *p* = 0.038) were significantly higher in the NC group compared to the MCI group.

At the genus level, the NC group exhibited significantly higher relative abundances of Faecalibacterium (LDA score = 4.456, *p* = 0.003) and Oscillospira (LDA score = 4.096, *p* = 0.002) compared to the MCI group. These genera are well-known for their SCFA-producing capabilities, particularly butyrate.

### Correlations between gut microbiota, BBB permeability, and cognitive function

Spearman correlation analysis revealed significant associations between the relative abundances of the depleted genera in the MCI group, hippocampal Ktrans values, and MoCA-B scores across all participants (*n* = 40). The relative abundance of Faecalibacterium was negatively correlated with hippocampal Ktrans (r = −0.466, *p* = 0.002) and positively correlated with MoCA-B scores (r = 0.596, *p* < 0.001). Similarly, the relative abundance of Oscillospira was negatively correlated with hippocampal Ktrans (r = −0.322, *p* = 0.043) and positively correlated with MoCA-B scores (r = 0.369, *p* = 0.019).

To investigate potential confounding by cardiometabolic factors, a new correlation analysis was performed between key cardiometabolic markers (SBP, DBP, Fasting Blood Glucose, LDL-C) and the relative abundances of Faecalibacterium and Oscillospira. No significant correlations were found (all *p* > 0.10), suggesting that the observed associations between these genera and cognitive/BBB measures are independent of these specific metabolic parameters within our cohort.

## Discussion

This study investigated the intricate relationships between gut microbiota composition, BBB permeability, and cognitive function in elderlies with CHD with and without MCI. Our principal findings demonstrate that elderlies with CHD and MCI exhibit significant gut dysbiosis, characterized by reduced microbial alpha diversity and distinct beta diversity profiles compared to their cognitively normal counterparts. Specifically, we identified a depletion of SCFA-producing bacteria, notably Faecalibacterium and Oscillospira, in the MCI group. Furthermore, these microbial alterations were associated with increased BBB permeability in the hippocampus and poorer cognitive performance. These results underscore the potential involvement of the gut-brain axis in the pathophysiology of cognitive decline in this high-risk population.

The observed reduction in gut microbial diversity (Chao1 and Shannon indices) in MCI patients aligns with findings from previous studies in non-CHD populations with MCI and AD (Gallo et al., 2024; Chen G. et al., 2023). Lower microbial diversity is often considered indicative of a less resilient and less functionally robust gut ecosystem (Hughes et al., 2001; Fassarella et al., 2021). Our beta diversity analysis further confirmed a significant structural divergence in the gut microbial communities of MCI patients, suggesting that specific compositional shifts, beyond mere diversity loss, are associated with cognitive status. It is important to note that since all participants had CHD, the observed differences in gut microbiota are more directly associated with the presence of MCI rather than CHD per se. CHD may act as a background factor that increases vulnerability, creating a state of systemic inflammation or vascular compromise where the effects of gut dysbiosis on the brain are amplified.

A key finding of our study is the depletion of SCFA-producing taxa, particularly Faecalibacterium and Oscillospira, in elderlies with CHD and MCI. Our focus on Faecalibacterium and Oscillospira is directly informed by our LEfSe results, which identified them as the most significantly depleted genera in the MCI group. Both genera are recognized for their capacity to produce butyrate, an SCFA with well-documented anti-inflammatory properties and a crucial role in maintaining gut barrier integrity and BBB function (Silva et al., 2020; Parker et al., 2020). Faecalibacterium, especially *Faecalibacterium prausnitzii*, is a prominent butyrate producer known to exert anti-inflammatory effects, partly by inhibiting the NF-κB pathway and promoting regulatory T-cell differentiation (Guo et al., 2024). Reductions in Faecalibacterium have been previously reported in MCI and AD cohorts and correlated with cognitive scores (Ueda et al., 2021). Its depletion could lead to compromised intestinal barrier function, potentially increasing the translocation of pro-inflammatory molecules like lipopolysaccharide (LPS) into systemic circulation, thereby contributing to chronic low-grade inflammation, BBB disruption, and neuroinflammation (Braniste et al., 2014; Loh et al., 2024).

Similarly, Oscillospira, another butyrate-producing bacterium, was found to be depleted in our MCI group. Recent studies suggest Oscillospira may play a protective role in neurodegenerative diseases, potentially by contributing to SCFA pools and modulating host inflammatory responses (Ma et al., 2025). The negative correlation we observed between these genera and BBB permeability (Ktrans), and their positive correlation with cognitive scores (MoCA-B), supports the hypothesis that a reduction in these beneficial bacteria may contribute to BBB dysfunction and is associated with cognitive decline. SCFAs, particularly butyrate, can directly enhance BBB integrity by upregulating the expression of tight junction proteins such as claudin-5 and occludin, and also by providing energy for colonocytes and modulating immune cell function (Silva et al., 2020; Martin-Gallausiaux et al., 2018; Parker et al., 2020). Reduced SCFA availability due to dysbiosis could therefore impair these protective mechanisms.

Our study also confirms that BBB breakdown, evidenced by increased hippocampal Ktrans values, is more pronounced in elderlies with CHD and MCI. This finding is consistent with previous research identifying BBB disruption as an early event in cognitive decline (Nation et al., 2019; Kaddoumi et al., 2022). The hippocampus, a brain region critical for learning and memory, is particularly vulnerable to damage in early AD. The observed correlation between higher Ktrans values and lower abundances of beneficial bacteria suggests a link where gut dysbiosis might contribute to BBB damage, possibly through reduced SCFA production and/or increased systemic inflammation, thereby facilitating cognitive impairment.

This study has several strengths, including the well-characterized cohort of elderlies with CHD, the use of standardized neuropsychological testing, and the direct measurement of BBB permeability using DCE-MRI alongside comprehensive gut microbiota analysis. However, certain limitations must be acknowledged. First, the cross-sectional design precludes definitive causal inferences; longitudinal studies are needed to establish temporality and causality between gut microbiota changes, BBB dysfunction, and cognitive decline. Second, the sample size (*n* = 40) is relatively modest, which may limit statistical power for some analyses, particularly for multivariable adjustments. Although we have now reported on comorbidities and key medication usages (e.g., statins, PPIs) and found them to be balanced between groups, residual confounding from other medications or unmeasured variables cannot be entirely excluded. A significant limitation is the lack of detailed dietary data, as diet is a primary driver of gut microbiota composition and an independent factor in cognitive health. Third, we used the Greengenes 13.8 database for taxonomic classification, which, while widely used, is no longer updated; future studies may benefit from using more current and comprehensive databases such as SILVA or GTDB. Furthermore, 16S rRNA gene sequencing provides information on microbial composition but does not directly assess functional capacity (e.g., actual SCFA production) or resolve to the strain level, which can have distinct functional implications. Future studies incorporating metagenomics, metabolomics (to measure fecal and serum SCFA levels), and culturomics would provide deeper insights. Fourth, BBB permeability was assessed only in the hippocampus; evaluating other brain regions might reveal more widespread or regionally specific BBB changes. Finally, while we focused on elderlies with CHD, the findings may not be generalizable to other populations with MCI.

Despite these limitations, our study provides valuable evidence linking gut dysbiosis, specifically the depletion of SCFA-producing bacteria like Faecalibacterium and Oscillospira, with increased BBB permeability and cognitive impairment in elderly individuals with CHD. These findings highlight the gut-brain axis as a potentially crucial modulatory pathway in the context of comorbid cardiovascular and neurocognitive disorders. Therapeutic strategies aimed at restoring gut microbial balance, such as targeted probiotics, prebiotics, dietary interventions, or fecal microbiota transplantation, could hold promise for mitigating BBB dysfunction and preserving cognitive function in this vulnerable aging population. Further research, particularly longitudinal and interventional studies, is warranted to validate these associations, elucidate the precise mechanisms involved, and explore the therapeutic potential of gut microbiota modulation in preventing or delaying cognitive decline in elderlies with CHD.

## Conclusion

In summary, this study demonstrates that elderly coronary heart disease individuals with mild cognitive impairment exhibit significant alterations in gut microbiota composition, including reduced diversity and a depletion of key SCFA-producing taxa such as Faecalibacterium and Oscillospira. These microbial changes are significantly correlated with increased blood–brain barrier permeability in the hippocampus and lower cognitive performance. These findings emphasize the potential contribution of gut dysbiosis to neurovascular dysfunction and cognitive decline in this specific high-risk population, suggesting that the gut-brain axis may serve as a promising target for future therapeutic interventions.

## Data Availability

The original contributions presented in the study are included in the article/Supplementary material, further inquiries can be directed to the corresponding author.

## Supplementary material

SUPPLEMENTARY FIGURE 1 Rarefaction curves evaluating sequencing depth. The plots illustrate alpha diversity, as measured by the Chao1 index (top), Shannon index (middle), and Pielou’s evenness index (bottom), plotted against the number of sampled sequences per sample. The curves represent the mean values for the Normal Cognition (NC; blue) and Mild Cognitive Impairment (MCI; red) groups, with error bars showing the standard deviation.

## References

[ref1] Albert M. S. DeKosky S. T. Dickson D. Dubois B. Feldman H. H. Fox N. C.. (2011). The diagnosis of mild cognitive impairment due to Alzheimer's disease: recommendations from the National Institute on Aging-Alzheimer's Association workgroups on diagnostic guidelines for Alzheimer's disease. Alzheimers Dement. 7, 270–279. doi: 10.1016/j.jalz.2011.03.008, PMID: 21514249 PMC3312027

[ref2] Bolyen E. Rideout J. R. Dillon M. R. Bokulich N. A. Abnet C. C. Al-Ghalith G. A.. (2019). Reproducible, interactive, scalable and extensible microbiome data science using QIIME 2. Nat. Biotechnol. 37, 852–857. doi: 10.1038/s41587-019-0209-9 31341288 PMC7015180

[ref3] Borrego-Ruiz A. Borrego J. J. (2024). Influence of human gut microbiome on the healthy and the neurodegenerative aging. Exp. Gerontol. 194:112497. doi: 10.1016/j.exger.2024.112497, PMID: 38909763

[ref4] Braniste V. Al-Asmakh M. Kowal C. Anuar F. Abbaspour A. Tóth M.. (2014). The gut microbiota influences blood-brain barrier permeability in mice. Sci. Transl. Med. 6:263ra158. doi: 10.1126/scitranslmed.3009759 PMC4396848 25411471

[ref5] Chen X. Zhang H. Ren S. Ding Y. Remex N. S. Bhuiyan M. S.. (2023). Gut microbiota and microbiota-derived metabolites in cardiovascular diseases. Chin. Med. J. 136, 2269–2284. doi: 10.1097/CM9.0000000000002206, PMID: 37442759 PMC10538883

[ref6] Chen G. Zhou X. Zhu Y. Shi W. Kong L. (2023). Gut microbiome characteristics in subjective cognitive decline, mild cognitive impairment and Alzheimer's disease: a systematic review and meta-analysis. Eur. J. Neurol. 30, 3568–3580. doi: 10.1111/ene.15961, PMID: 37399128

[ref7] Cryan J. F. O'Riordan K. J. Cowan C. S. M. Sandhu K. V. Bastiaanssen T. F. S. Boehme M.. (2019). The microbiota-gut-brain Axis. Physiol. Rev. 99, 1877–2013. doi: 10.1152/physrev.00018.2018, PMID: 31460832

[ref8] Deckers K. Schievink S. H. J. Rodriquez M. M. F. van Oostenbrugge R. J. van Boxtel M. P. J. Verhey F. R. J.. (2017). Coronary heart disease and risk for cognitive impairment or dementia: systematic review and meta-analysis. PLoS One 12:e0184244. doi: 10.1371/journal.pone.0184244, PMID: 28886155 PMC5590905

[ref9] Fassarella M. Blaak E. E. Penders J. Nauta A. Smidt H. Zoetendal E. G. (2021). Gut microbiome stability and resilience: elucidating the response to perturbations in order to modulate gut health. Gut 70, 595–605. doi: 10.1136/gutjnl-2020-321747, PMID: 33051190

[ref10] Gallo A. Martone A. M. Liperoti R. Cipriani M. C. Ibba F. Camilli S.. (2024). Mild cognitive impairment and microbiota: what is known and future perspectives. Front. Med. 11:1410246. doi: 10.3389/fmed.2024.1410246, PMID: 38957302 PMC11217486

[ref11] Guo J. Meng F. Hu R. Chen L. Chang J. Zhao K.. (2024). Inhibition of the NF-κB/HIF-1α signaling pathway in colorectal cancer by tyrosol: a gut microbiota-derived metabolite. J. Immunother. Cancer 12:e008831. doi: 10.1136/jitc-2024-008831, PMID: 39343509 PMC11440206

[ref12] Hughes J. B. Hellmann J. J. Ricketts T. H. Bohannan B. J. (2001). Counting the uncountable: statistical approaches to estimating microbial diversity. Appl. Environ. Microbiol. 67, 4399–4406. doi: 10.1128/AEM.67.10.4399-4406.2001, PMID: 11571135 PMC93182

[ref13] Jia L. Du Y. Chu L. Zhang Z. Li F. Lyu D.. (2020). Prevalence, risk factors, and management of dementia and mild cognitive impairment in adults aged 60 years or older in China: a cross-sectional study. Lancet Public Health 5, e661–e671. doi: 10.1016/S2468-2667(20)30185-7, PMID: 33271079

[ref14] Kaddoumi A. Denney T. S. Jr. Deshpande G. Robinson J. L. Beyers R. J. Redden D. T.. (2022). Extra-virgin olive oil enhances the blood-brain barrier function in mild cognitive impairment: a randomized controlled trial. Nutrients 14:5102. doi: 10.3390/nu14235102, PMID: 36501136 PMC9736478

[ref15] Kulkarni R. Kumari S. Dhapola R. Sharma P. Singh S. K. Medhi B.. (2025). Association between the gut microbiota and alzheimer’s disease: an update on signaling pathways and translational therapeutics. Mol. Neurobiol. 62, 4499–4519. doi: 10.1007/s12035-024-04545-2, PMID: 39460901

[ref16] Loh J. S. Mak W. Q. Tan L. K. S. Ng C. X. Chan H. H. Yeow S. H.. (2024). Microbiota-gut-brain axis and its therapeutic applications in neurodegenerative diseases. Signal Transduct. Target. Ther. 9:37. doi: 10.1038/s41392-024-01743-1, PMID: 38360862 PMC10869798

[ref17] Ma X. Liu J. Jiang L. Gao Z. Shi Z. Zhang N.. (2025). Dynamic changes in the gut microbiota play a critical role in age-associated cognitive dysfunction via SCFAs and LPS synthesis metabolic pathways during brain aging. Int. J. Biol. Macromol. 304:140945. doi: 10.1016/j.ijbiomac.2025.140945, PMID: 39947548

[ref18] Martin-Gallausiaux C. Béguet-Crespel F. Marinelli L. Jamet A. Ledue F. Blottière H. M.. (2018). Butyrate produced by gut commensal bacteria activates TGF-beta1 expression through the transcription factor SP1 in human intestinal epithelial cells. Sci. Rep. 8:9742. doi: 10.1038/s41598-018-28048-y, PMID: 29950699 PMC6021401

[ref19] Nation D. A. Sweeney M. D. Montagne A. Sagare A. P. D’Orazio L. M. Pachicano M.. (2019). Blood-brain barrier breakdown is an early biomarker of human cognitive dysfunction. Nat. Med. 25, 270–276. doi: 10.1038/s41591-018-0297-y, PMID: 30643288 PMC6367058

[ref20] Parker A. Fonseca S. Carding S. R. (2020). Gut microbes and metabolites as modulators of blood-brain barrier integrity and brain health. Gut Microbes 11, 135–157. doi: 10.1080/19490976.2019.1638722, PMID: 31368397 PMC7053956

[ref21] Petersen R. C. Lopez O. Armstrong M. J. Getchius T. S. D. Ganguli M. Gloss D.. (2018). Practice guideline update summary: mild cognitive impairment [RETIRED]: report of the guideline development, dissemination, and implementation subcommittee of the American Academy of Neurology. Neurology 90, 126–135. doi: 10.1212/WNL.0000000000004826 29282327 PMC5772157

[ref22] Prajapati S. K. Wang S. Mishra S. P. Jain S. Yadav H. (2025). Protection of Alzheimer's disease progression by a human-origin probiotics cocktail. Sci. Rep. 15:1589. doi: 10.1038/s41598-024-84780-8, PMID: 39794404 PMC11724051

[ref23] Silva Y. P. Bernardi A. Frozza R. L. (2020). The role of short-chain fatty acids from gut microbiota in gut-brain communication. Front. Endocrinol. 11:25. doi: 10.3389/fendo.2020.00025, PMID: 32082260 PMC7005631

[ref24] Sperling R. A. Aisen P. S. Beckett L. A. Bennett D. A. Craft S. Fagan A. M.. (2011). Toward defining the preclinical stages of Alzheimer's disease: recommendations from the National Institute on Aging-Alzheimer's Association workgroups on diagnostic guidelines for Alzheimer's disease. Alzheimers Dement. 7, 280–292. doi: 10.1016/j.jalz.2011.03.003, PMID: 21514248 PMC3220946

[ref25] Spielman L. J. Gibson D. L. Klegeris A. (2018). Unhealthy gut, unhealthy brain: the role of the intestinal microbiota in neurodegenerative diseases. Neurochem. Int. 120, 149–163. doi: 10.1016/j.neuint.2018.08.005, PMID: 30114473

[ref26] Tang W. H. Kitai T. Hazen S. L. (2017). Gut microbiota in cardiovascular health and disease. Circ. Res. 120, 1183–1196. doi: 10.1161/CIRCRESAHA.117.309715, PMID: 28360349 PMC5390330

[ref27] Ueda A. Shinkai S. Shiroma H. Taniguchi Y. Tsuchida S. Kariya T.. (2021). Identification of *Faecalibacterium prausnitzii* strains for gut microbiome-based intervention in Alzheimer's-type dementia. Cell Rep. Med. 2:100398. doi: 10.1016/j.xcrm.2021.100398, PMID: 34622235 PMC8484692

[ref28] Wang Q. Huang X. Su Y. Yin G. Wang S. Yu B.. (2022). Activation of Wnt/β-catenin pathway mitigates blood-brain barrier dysfunction in Alzheimer's disease. Brain 145, 4474–4488. doi: 10.1093/brain/awac236 35788280 PMC9762951

