# Supplementary figures and images for: Gut dysbiosis is associated with increased blood–brain barrier permeability and cognitive impairment in elderlies with coronary heart disease
Source: Front Aging Neurosci. 2025 Aug 13;17:1640761. doi: 10.3389/fnagi.2025.1640761 (PMC12380634; doi:10.3389/fnagi.2025.1640761)

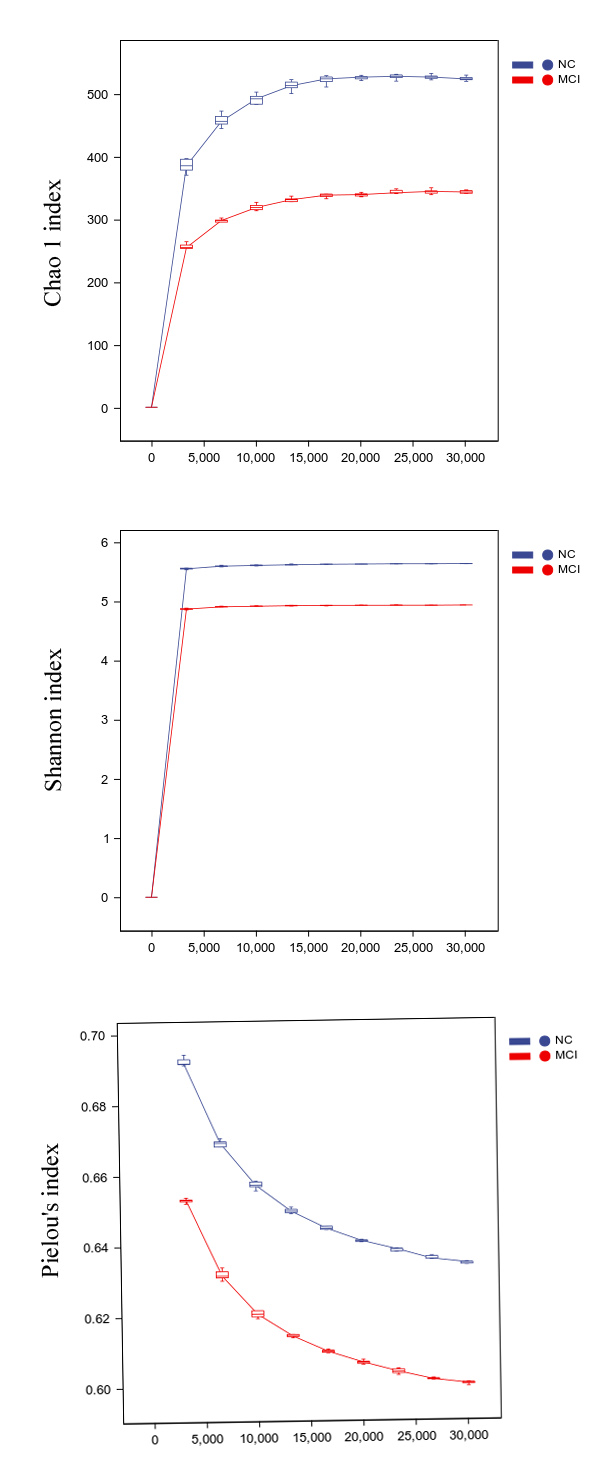

Supplement: SUPPLEMENTARY FIGURE 1 — Rarefaction curves evaluating sequencing depth. The plots illustrate alpha diversity, as measured by the Chao1 index (top), Shannon index (middle), and Pielou’s evenness index (bottom), plotted against the number of sampled sequences per sample. The curves represent the mean values for the Normal Cognition (NC; blue) and Mild Cognitive Impairment (MCI; red) groups, with error bars showing the standard deviation. [file Image_1.jpeg]
